# Crowdsourced Curriculum Development for Online Medical Education

**DOI:** 10.7759/cureus.1925

**Published:** 2017-12-08

**Authors:** Eric Shappell, Teresa M Chan, Brent Thoma, N Seth Trueger, Bob Stuntz, Robert Cooney, James Ahn

**Affiliations:** 1 Section of Emergency Medicine, University of Chicago; 2 Faculty of Health Sciences, Department of Medicine, Division of Emergency Medicine, McMaster University; 3 Department of Emergency Medicine, College of Medicine, University of Saskatchewan; 4 Emergency Medicine, Northwestern University Feinberg School of Medicine; 5 Emergency Medicine, Wellspan York Hospital; 6 Department of Emergency Medicine, Geisinger Health System

**Keywords:** free open access medical education, crowdsourcing, curriculum development, online education, curriculum design, needs assessment, emergency medicine, curriculum, crowd-sourcing, curriculum evaluation

## Abstract

In recent years online educational content, efforts at quality appraisal, and integration of online material into institutional teaching initiatives have increased. However, medical education has yet to develop large-scale online learning centers. Crowd-sourced curriculum development may expedite the realization of this potential while providing opportunities for innovation and scholarship. This article describes the current landscape, best practices, and future directions for crowdsourced curriculum development using Kern’s framework for curriculum development and the example topic of core content in emergency medicine.

A scoping review of online educational content was performed by a panel of subject area experts for each step in Kern’s framework. Best practices and recommendations for future development for each step were established by the same panel using a modified nominal group consensus process.

The most prevalent curriculum design steps were (1) educational content and (2) needs assessments. Identified areas of potential innovation within these steps included targeting gaps in specific content areas and developing underrepresented instructional methods. Steps in curriculum development without significant representation included (1) articulation of goals and objectives and (2) tools for curricular evaluation.

By leveraging the power of the community, crowd-sourced curriculum development offers a mechanism to diffuse the burden associated with creating comprehensive online learning centers. There is fertile ground for innovation and scholarship in each step along the continuum of curriculum development. Realization of this paradigm’s full potential will require individual developers to strongly consider how their contributions will align with the work of others.

## Introduction and background

Online education is rapidly expanding across many disciplines, and graduate medical education is no exception [1-6]. Disruptive innovations such as massive open online courses have been heralded as pillars in a future paradigm of collaborative online learning for medical education [7]. While in recent years we have seen an increase in online educational content, quality appraisal initiatives [8-13], and integration of online material into institutional teaching initiatives [9, 14], medical education remains far from realizing large-scale online learning centers such as those described by Mehta, et al. [7].

One clear barrier to making this vision a reality is the enormous amount of work involved in developing such comprehensive resources. While many have succeeded in creating a wide variety of high-quality online educational resources, no single or curated collection of open-access resources boasts the scope to cover an entire subject, specialty, or discipline. Strides are being made in both the creation and curation of content, however, and the potential for this type of comprehensive resource is real [9, 14, 15]. While this progress is encouraging, it is important to note that educational content is only one piece of the puzzle and another less discussed but significant barrier remains: the integration of educational content into a purposeful curriculum.

Rigorous curriculum design is not foreign to the realm of open-access online education; there are many examples of successful small-scale curricular interventions that have used this platform [16-19]. However, examples of large-scale curricular interventions are lacking. Despite the lack of current examples, evidence of a movement toward the formation of such resources is mounting as multiple independent sources develop components of traditional curriculum design for large-scale online curricula [9, 14, 20, 21].

With multiple individuals contributing to curriculum development, the education community stands to benefit from relief of the burden of completing each curriculum design step alone. This “crowdsourced curriculum development” is an exciting prospect for medical educators interested in robust online curriculum design and provides opportunities for scholarship throughout the continuum of curriculum creation. In this article, through a review of online medical education resources and consensus process for establishing recommendations, we will illustrate current examples and future directions for crowdsourced curriculum development using Kern’s framework and the topic of core content in emergency medicine [22].

## Review

The scoping review process was conducted by an expert panel composed of the authors, each of whom possesses knowledge and experience in the realm of online medical education. Each step in Kern’s framework (problem identification, needs assessment, goals and objectives, educational strategies, implementation, measuring outcomes) was explored individually. The search process was conducted from September to November of 2016. All authors participated in the search process. The search included a scientific database (PubMed) and a general search engine (Google). Search terms including the titles of each of Kern's steps, 'emergency medicine,' 'intern,' 'curriculum,' 'core content,' 'FOAM,' and 'FOAMed.' In addition, resources known to the authors and those identified by snowball sampling were included.

A modified nominal group consensus process was used to determine the final list of resources and recommendations for further development of each step. The consensus process continued until all authors agreed on the final list of resources to be included. This study did not involve human subjects.

Step 1: Problem identification

Purpose

In Kern’s model, the “problem” to be identified may be thought of as the difference between the way something is currently taught and the ideal solution [22].

“The better a problem is defined, the easier it will be to design an appropriate curriculum to address the problem.” (Kern, p. 11)

This process of problem analysis is also referred to as a general needs assessment.

Example

In the example of a curriculum on core content for emergency medicine, the most common model relies on didactic sessions, required textbook readings, and on-shift teaching. These traditional education methods have well-described weaknesses: didactic sessions suffer from variable attendance (due to off-service rotations and other clinical and wellness obligations) [23], millennial residents have largely eschewed the textbook [21, 24, 25], and on-shift teaching is highly dependent on patient pathology and lacks the consistency to assure a suitable foundation in core concepts [26-28]. These limitations could be addressed with an online curriculum that is always available, utilizes educational methods that the learners value, and is designed with the rigor and consistency required to assure a suitable foundation in core concepts.

Recommendations

Educators should explicitly define the problems that educational interventions are designed to address. This step is largely under-represented in the paradigm of large-scale online medical education. Educators interested in advancing this paradigm may begin by clearly articulating a problem that is used to align stakeholders and assimilate resources.

Step 2: Targeted needs assessment

Purpose

The targeted needs assessment begins with the identification of target learners (defined as “the group most likely, with further learning, to contribute to the solution of the problem” [22]), followed by an evaluation of the specific needs of these learners relative to the identified problem.

Example

In our example of a curriculum for core content in emergency medicine, the target population would include emergency medicine residents and senior medical students bound for a career in emergency medicine. Those responsible for emergency medicine training programs and certifying bodies would be crucial stakeholders to include in the targeted needs assessment. To assess the needs of this broad and diverse population, multiple assessments for each stakeholder group may be required.

There are several existing examples of targeted needs assessments that align with a web-based curriculum on core content in emergency medicine outlined in Table 1.

**Table 1:** Needs assessments for core content in emergency medicine.

Source	Description
Emergency medicine clerkship curriculum: an update and revision [29, 30]	Consensus process used to develop online curriculum for fourth year medical students on their emergency medicine clerkship
An Evaluation of Emergency Medicine Core Content Covered by Free Open Access Medical Education Resources [20]	Identified lack of alignment between online resources and the American Board of Emergency Medicine's Model of the Clinical Practice of Emergency Medicine
A Needs Assessment for a Longitudinal Emergency Medicine Intern Curriculum [21]	Regional survey of emergency medicine outlining learners’ perceptions of essential topics to cover in the first year of training
Targeted Needs Assessment of Off-service Residents in Emergency Medicine [31]	Survey of internal medicine residents regarding areas for improvement in the preparation of off-service rotators for addressing core concepts in emergency medicine
Bleeding and Clotting: A SoMe-based Needs Assessment [32]	Social media-based needs assessment developed to drive content production related to core concepts in bleeding and clotting

Recommendations

Existing targeted needs assessments may be applicable to large-scale online medical education; however, confounding factors in the translation of existing evidence to this new paradigm must be addressed. Formulation of a large-scale needs assessment (either de novo or through assimilation and adaptation of existing resources) would meaningfully contribute to the realization of this new paradigm. Once large-scale needs assessments are conducted, local education stakeholders may wish to conduct additional needs assessments at the program level to determine whether the curriculum appropriately addresses the needs of local learners.

Step 3: Goals and objectives

Purpose

Goals provide a broad overview of the intent of the curriculum. Objectives provide specific and measurable educational directives that build toward the broad educational goal of the curriculum. These constructs communicate the purpose and proper usage of the curriculum, in addition to informing the design of assessments (see Step 6). The importance of this step is reflected in the Accreditation Council for Graduate Medical Education (ACGME) common program requirement that curricula include “Competency-based goals and objectives for each assignment at each educational level” [33].

Example

Unfortunately, despite the large amount of quality online educational content, rarely are resources accompanied by goals and objectives that clarify the desired outcomes from use of the resource. We identified two online resources that consistently outline goals and objectives for online core content in emergency medicine: EM Fundamentals [34] and the Clerkship Directors in Emergency Medicine (CDEM) website [29].

Recommendations

Content generated without a clear measure of learner success is of limited use to instructors and leaves self-assessing learners directionless. Clearly outlined goals and objectives provide transparency across stakeholder groups and a benchmark for measurement of learners.

Writing clear objectives can be a difficult task for curriculum designers. We suggest using the SMART framework to assist when designing educational objectives [35]. These criteria for objectives were originally described in business literature and have been adapted to medical education (Table 2). When using the SMART framework, all objectives must hold these four elements: 1) “Who will do,” 2) “How much or how well,” 3) “Of what” and 4) “By when?”

**Table 2:** SMART framework for designing educational objectives. Adapted from [35].

SMART Framework	Definition
Specific	The desired result should be specifically mentioned. Generalities and vague terms should be avoided.
Measurable	The specific result should be measurable. Outcomes that cannot be feasibly measured should be avoided. The more difficult the objective is to measure, the more difficult it will be to assess whether the curriculum achieved its goal.
Achievable	The learner must be capable of achieving the desired result.
Realistic	Consider time, resource, and other constraints to determine if an achievable goal is realistic. It should be reasonable to expect the learner to complete the objective within the confines of their situation.
Time-based	All objectives should specify by when the learner should hit the specific and measurable target(s).

Goals and objectives are intimately linked to educational strategies and assessment, therefore developing these steps in parallel will help ensure alignment. Existing content need not be overhauled by originators to include this language; goals and objectives for high-quality content can be crowdsourced. Mapping existing content to nationally recognized goals and objectives (e.g., those of specialty organizations, ACGME competencies and/or milestones) will likely be more interesting to outside stakeholders than retrofitting content to custom goals and objectives.

Step 4: Educational strategies

Purpose

Educational strategies are the vehicle for learner progression. Multiple methods may be used to reinforce a single concept or to target different levels of objectives within a single curriculum.

Example

To date, the ad hoc nature of online content production has, unsurprisingly, led to an imbalance in coverage of various topics [20]. However, a small but growing number of resources have focused on foundational knowledge (Table 3).

**Table 3:** Online resources featuring core content in emergency medicine.

Resource	Content Type(s)	Website
ALiEM AIR	Curated online resources, quizzes	https://www.aliem.com/aliem-approved-instructional-resources-air-series/
CoreEM	Blog, podcast, videos	https://coreem.net/
CrackCast on CanadiEM.org	Question and answer blog posts, Podcast, Flashcards	https://canadiem.org/crackcast/
EM Basic	Blog, podcast	http://embasic.org/
EM Foundations	Curated textbook and online resources, flipped classroom resources	http://emergencymedicinefoundations.com
EM Fundamentals	Curated online resources and primary literature, flipped classroom resources	http://emfundamentals.com/
EMSimCases	Simulation cases	https://emsimcases.com

These foundationally-focused resources are mostly text- and video-based, which are reasonable methods for objectives pertaining to basic knowledge. While most blogs permit some level of interaction through comments and responses, objectives related to higher-level functions (such as application and analysis) may be better targeted using social media tools as forums for discussion (e.g., Twitter, Slack, Google hangouts, Reddit). If classroom time is available, higher-level functions may also be targeted by using a blended or flipped classroom approach, leveraging online content for preparation and interactive small group activities for the live sessions.

Recommendations

While online educational content is arguably the most developed step in Kern’s framework at present, significant gaps still exist. Educators motivated to advance this area should focus on underrepresented content areas and/or underrepresented methods of instruction (e.g., interactive online experiences).

Step 5: Implementation

Purpose

The implementation phase focuses on the logistics of resource development, deployment, and sustainability.

Example

There are two ways in which educators deliver online content: (1) contributing to an existing resource and (2) implementing a new online resource. The simplest way to deliver content is by contributing to an existing resource with an established product and reputation. This process is akin to journal submissions: many existing web resources will review submitted content and, if deemed to meet an appropriate standard, publish the submission. Existing resources that allow third-party submissions include CanadiEM [36], EMDocs [37], EMSimCases [38], and R.E.B.E.L. EM [39].

There are also many formats for deployment of new online resources, each with variable start-up costs and diverse hosting options. For podcasts, start-up costs may include a microphone, audio processing software, and a hosting service (e.g., Libsyn, Soundcloud). For video creation, costs may include a camera and video processing software. Free video hosting services include YouTube and Vimeo. For websites, costs may include hosting fees, web development software, and potentially hiring professional web developers. There are also many options for web hosts, some of which are free (e.g., Wordpress.com, Wix, Blogger). Each of these steps also carries non-financial requirements (e.g., basic technical skills).

In addition to start-up costs, financial support to enable upkeep will be necessary. Currently used models for ongoing financial support include local and national organizational funding [34, 40, 41], ad revenue, the freemium model [42], and subscription services [43].

Recommendations

Creating a new web resource can require significant investments of time and money. New resources will also need to build a reputation with learners. When starting out, consider modeling after a known successful resource or contributing to an existing host in order to conserve resources while gaining experience. New resources should include a sustainability plan that addresses sources of funding for continuing costs.

Step 6: Measuring outcomes (assessment and evaluation)

Purpose

Assessment of learners and evaluation of usefulness are the methods by which outcomes data pertaining to the curriculum are obtained. Without robust mechanisms for assessment and evaluation, educators will lack meaningful information regarding the successes or shortcomings of the educational intervention. Data from these steps may be used to highlight areas for improvement in the iterative process of curriculum development.

Example: Assessment of Learning

Arguably the most robust online resource for assessment of learners on core content in emergency medicine is the suite developed by Academic Life in Emergency Medicine (ALiEM) [41]. These include ALiEMU, ALiEM Capsules, ALiEM AIR, and ALiEM AIR PRO, which track the learner’s progress using multiple-choice quizzes. ALiEM also hosts the Medical Education in Cases (MEdIC) series, in which participants contribute to an open discussion forum via blog comments [44]. This format allows instructors to monitor uptake and understanding of a topic. An existing tool for self-assessment is the flashcard bank developed by the CRACKCast podcast [45]. Finally, there are several online resources that could be used to facilitate learner assessment at the local level. EM Sim Cases is an online repository of peer-reviewed simulation cases that are presented in a standardized format [46], and the McMaster Modular Assessment Program (McMAP) has three e-books that contain numerous competency-based assessment tools [47]. Online question banks may also be used locally as readiness assurance tests in the flipped-classroom model.

Example: Evaluation of Usefulness

Our search of existing resources has found that robust evaluation tools are lacking. Most online resources provide contact information for the authors that may be used to submit feedback as a form of evaluation, however, this mechanism is inefficient and unreliable. Participation as a proxy for usefulness, however, can be easily quantified in the online environment. For websites, it is possible to embed code that measures usage with metrics such as page views, unique users, and time the users spent on a specific page. For podcasts, it is possible to track analytics such as number of downloads and subscribers. An amalgam of metrics has been developed in an effort to measure website impact [48]. It must be noted, however, that these metrics only measure engagement with the resource and are limited in their ability to truly measure usefulness.

Recommendations

**Figure 1:**
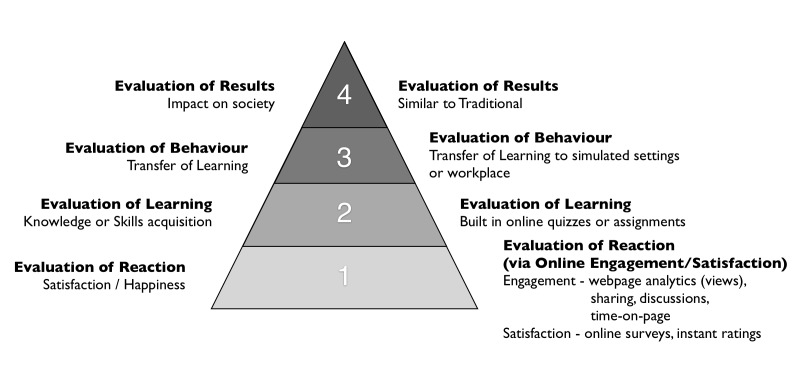
Kirkpatrick Framework re-imagined for the online world. Adapted from [49].

For the online environment, we propose educators use an adaptation of Kirkpatrick’s framework for program evaluation (Figure 1) [49]. The dearth of robust mechanisms for formal evaluation of online resources as curricular tools makes this another area ripe for innovation and scholarship. Table 4 outlines recommendations for gathering and communicating evaluation data in the absence of more robust intrinsic evaluation tools.

**Table 4:** Gathering and communicating evaluations and feedback.

	Gather Evaluations	Communicate Feedback
Timing	After learners use the materials	After reviewing evaluation data
Goal	Gather information from learners regarding usefulness of selected resources, including: Assessment of fitness Notation of points of controversy Identification of errors and translational issues (e.g., imperial vs. SI units) Suggestions for alternate resources	Provide feedback to the creators of the work, including: Applicability to curriculum Areas of strength Areas of controversy Identified gaps Identified errors and translational issues
Methods	Survey learners for feedback (n.b. if conducting a flipped classroom, consider gathering feedback immediately upon arrival prior to potential priming and while material fresh). Some questions to ask when creating your evaluation: Did you find the resource’s content appropriate given the goals and objectives for this session? If not, please describe the shortcoming. Do you have suggestions for how to improve this resource? Were the materials easy to access and well-designed? If not, please comment on areas for improvement. Did you have time to complete the resources?	Public Feedback: Comment and Tweet For many resources there are ways to provide post-publication feedback in a public forum (e.g., via the comments section of the web forum or on a social media platform such as Twitter). This venue not only benefits the creator, but also community members who can appreciate the example context in which the resource is being used (i.e., your curriculum) as well as the strengths and weaknesses identified when utilizing the resource for this purpose. Private Feedback: Email/Contact the Author Most repositories and content producers will list a method to privately contact the authors. This method is suggested for potentially sensitive feedback.

Summary

Development of comprehensive online learning experiences is a daunting task. This approach has been successful in other fields, however, and medical education stands to similarly benefit from this model. Crowdsourced curriculum development offers a mechanism to diffuse the burden associated with realization of this promising new paradigm. While this article has used the topic example of core content in emergency medicine to review the state of online resources relative to Kern’s framework, the same principles can be applied to any number of topics. As described above, this method of curriculum design begins with the notion that not all scholarly contributions to the field need to include new educational resources. Rather, there is fertile ground for novel scholarship in each step along the continuum of curriculum development and in curating existing resources to align with a specific educational initiative.

We note that some of Kern’s steps in curriculum development are already well-represented, including educational content. Many existing problems and needs assessments may align with these existing resources. Innovation in this area is still possible and may target gaps in content or instructional methods, or perhaps synthesize existing material to note or address a specific need.

Underrepresented steps in Kern’s framework include the articulation of goals and objectives and tools for curricular evaluation. Each of these areas is clear targets for educators interested in contributing scholarship to advance this new paradigm.

This study limited focus to the example topic of core content in emergency medicine. It is possible that the existing landscape in other content areas varies significantly; however, the approach to curriculum development in these areas would remain the same. Also, while this study used multiple modalities to identify content relevant to each of the highlighted areas (e.g., scholarly search engines, general search engines, and snowball sampling), it is possible that some relevant resources that are earlier in their development were still not discovered. As these resources mature and gain prominence, they may play important roles in the framework described.

## Conclusions

By leveraging the power of the community, crowd-sourced curriculum development offers a mechanism to diffuse the burden associated with creating comprehensive online learning centers. There is fertile ground for innovation and scholarship in each step along the continuum of curriculum development. Realization of this paradigm’s full potential will require individual developers to consider how their contributions will align with the work of others to be most impactful for the community.
